# *Senecio festucoides* (Senecioneae, Compositae), a new species from northern Chile

**DOI:** 10.3897/phytokeys.149.52297

**Published:** 2020-06-03

**Authors:** Joel Calvo, Andrés Moreira-Muñoz

**Affiliations:** 1 Instituto de Geografía, Facultad de Ciencias del Mar y Geografía, Pontificia Universidad Católica de Valparaíso, Avenida Brasil 2241, 2362807, Valparaíso, Chile Pontificia Universidad Católica de Valparaíso Valparaíso Chile

**Keywords:** Andes, Asteraceae, dichotomous key, taxonomy

## Abstract

*Senecio festucoides* is described from northern Chile. The new species is morphologically similar to the discoid caespitose Andean species and belongs to the subgroup displaying yellow corollas and yellowish anthers and style branches. It is characterized by a weak, not self-supporting stem, narrowly linear leaves, long pedunculate capitula with (17–)21 involucral bracts, and minutely papillose achenes. Among other characters, the color of the corollas, anthers, and style branches and the number of involucral bracts differentiate it from *S. scorzonerifolius*, which is the morphologically closest species. The new species thrives in the desertic Puna ecoregion and grows amongst tufts of *Festuca chrysophylla* (Poaceae). Detailed pictures of living plants are provided, as well as a distribution map and a dichotomous key to the discoid caespitose *Senecio* species from northern Chile.

## Introduction

*Senecio* L. (Compositae) is one of the largest genera of flowering plants harboring ca. 1250 species (Pelser et al. 2007; Nordenstam et al. 2009). It has a nearly cosmopolitan distribution with a remarkable diversification in Mediterranean climate zones, i.e. South Africa, Chile, and the Mediterranean Basin (Calvo et al. 2015).

In Chile, the first comprehensive revision of the genus recognized 208 species and treated 11 taxa as dubious (Cabrera 1949). Some of the species accepted by Cabrera are currently placed in new or resurrected genera as a result of efforts to redefine the generic delimitation of *Senecio* (Nordenstam et al. 2009). This is the case for *S. yegua* (Colla) Cabrera and *S. cymosus* J.Rémy, which are widely accepted as being part of the genus *Acrisione* B.Nord. Likewise, traditionally accepted genera such as *Culcitium* Bonpl. and *Robinsonia* DC. are currently treated within *Senecio* in order to move towards a monophyletic generic concept of this genus (Pelser et al. 2007; Pelser et al. 2010). Further contributions aimed at improving the understanding of the Chilean species mainly concerned nomenclatural adjustments (Marticorena and Quezada 1974; Soldano 1998) and the addition of new records and species (Cabrera 1954, 1955; Ricardi and Marticorena 1964; Marticorena and Quezada 1974, 1978; Moreira-Muñoz et al. 2016; Muñoz-Schick et al. 2016; Rodríguez et al. 2018; Calvo and Moreira-Muñoz 2019; Calvo et al. 2019). The species described in Chile after the taxonomic revision by Cabrera (1949) are detailed, and taxonomically updated if needed, in Table 1.

The checklist of the Chilean flora records 233 *Senecio* species (Rodríguez et al. 2018), however, this is an estimated number that will require adjusting following further taxonomic revision of the group. Here, we describe a new species from northern Chile as a result of field work carried out during 2019 and 2020. It is similar to discoid caespitose species and belongs to the subgroup displaying yellow corollas and yellowish anthers and style branches (see Calvo et al. 2019). The morphologically closest species is *S. scorzonerifolius* Meyen & Walp. A detailed taxonomic discussion, a distribution map, and pictures of living plants are provided, as well as a dichotomous key to the discoid caespitose *Senecio* species from northern Chile.

**Table 1.** Species of *Senecio* described from Chile after the taxonomic revision by Cabrera (1949).

Species Name	Publication Year	Status
*Senecio behnii* Ricardi & Martic.	1964	accepted
*Senecio coscayanus* Ricardi & Martic.	1964	accepted
*Senecio guatulamensis* Muñoz-Schick, A.Moreira & Trenq.	2016	accepted
*Senecio jilesii* Cabrera	1955	accepted
*Senecio laucanus* Ricardi & Martic.	1964	= *Senecio pygmophyllus* (S.F.Blake) J.Calvo, A.Granda & V.A.Funk
*Senecio munnozii* Cabrera	1954	accepted
*Senecio olivaceobracteatus* Ricardi & Martic.	1964	= *Senecio tacorensis* Cabrera
*Senecio pappii* Ricardi & Martic.	1964	accepted
*Senecio pfisteri* Ricardi & Martic.	1964	= *Xenophyllum esquilachense* (Cuatrec.) V.A.Funk
*Senecio ricardii* Martic. & Quezada	1974	accepted
*Senecio socompae* Cabrera	1954	accepted
*Senecio toconaoensis* J.Calvo & A.Moreira	2019	accepted
*Senecio zapahuirensis* Martic. & Quezada	1978	accepted

## Material and methods

This contribution is the result of bibliographic review, field work in northern Chile, and the revision of specimens kept at BOLV, CONC, LPB, and SGO. Additionally, a few digital specimens from K, LP, and US were studied; herbarium acronyms follow Thiers (2020).

## Taxonomy

### Senecio festucoides

Taxon classificationPlantaeAsteralesAsteraceae

J.Calvo & A.Moreira sp. nov.

F93E9EA8-D513-5C15-9F6D-8E503E4040E8

urn:lsid:ipni.org:names:77209838-1

Figs 1 , 2A–C

#### Diagnosis.

The new species mainly differs from its morphologically closest species *S. scorzonerifolius* in the yellow corollas, yellowish anthers and style branches, glabrous leaves, long peduncles with 1–3 linear bracts, and by having (17–)21 involucral bracts.

#### Type.

**Chile.** Antofagasta: San Pedro de Atacama, Machuca, 4 km antes de la entrada a los géisers del Tatio, 22°23'S, 68°1'W, 4375 m, 27 Feb 2020, fl. and fr., *J. Calvo 8120* (holotype: SGO; isotypes: CONC, MA, US).

Perennial not self-supporting herb, rarely decumbent, with a thin rhizome. ***Stem*** 15–25 cm tall, 1.8–2.5 mm wide, rather terete, branched in the lower part, weak. ***Leaves*** alternate, narrowly linear (leaf width/length ratio 0.01−0.03), 41–78 mm long, 0.9–1.2 mm wide, apex acute, base sessile (uniform in width), margin entire and flat (rarely with a few distant teeth), elliptic in cross section (rather flat when dried), glabrous on both surfaces, yellowish green, with a graminoid appearance. ***Capitula*** discoid, solitary, terminal, pedunculate; peduncle 5–6 cm long, glabrous, with 1–3 linear bracts up to 6 mm long. ***Involucre*** 12–14 mm long, 8–10 mm wide (in living plants); involucral bracts (17–)21, oblong-lanceolate, 10–11 mm long, 1.2–2 mm wide, acute to attenuate at the apex, smooth, glabrous, blackish-tipped; supplementary bracts (3–) 5–8, linear, 3.9–6 mm long, 0.9–1.1 mm wide, smooth, a third as long as the involucral bracts, glabrous, blackish-tipped. ***Florets*** 85–95, hermaphrodite; corolla tubular, 7.5–8.8 mm long, 0.5–0.7 mm wide, 5-lobed, the limb usually longer than the tube, yellow. Anthers yellowish; anther appendages 2–3 times longer than wide, ca. 0.5 × 0.2 mm; anther bases auriculate; filament collar balusterform. Style branches truncate with a crown of sweeping hairs, yellowish. ***Achenes*** 4.2–5.3 mm long, 0.9–1 mm wide, 8–10 ribbed, minutely papillose, brownish; pappus 7.5–9 mm long, barbellate, whitish. Chromosome number: unknown.

#### Distribution and habitat.

Chile (Antofagasta, Tarapacá). Considering the proximity of both populations to the Bolivian territory and the presence of similar environments across the border, its presence in this latter country is likely. It thrives in exposed grassy slopes and plains of the desertic Puna ecoregion, between elevations of 4325‒4550 m (Fig. 3).

**Figure 1.**
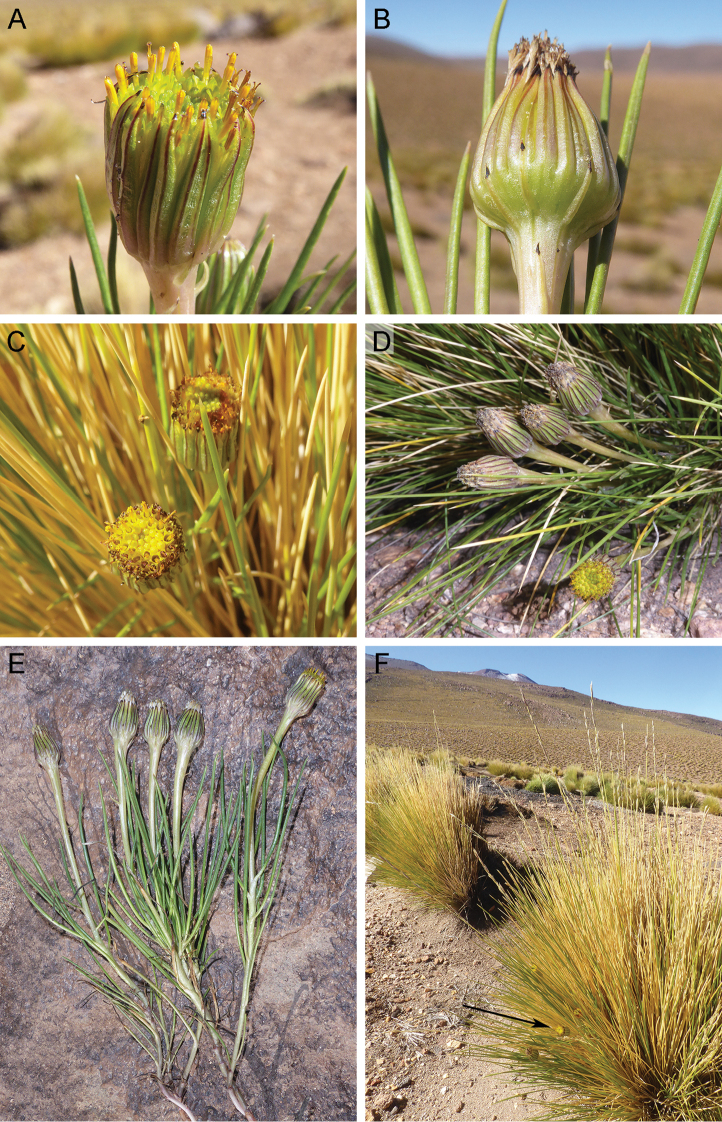
*Senecio festucoides***A** involucral bracts and florets **B** supplementary bracts **C, D** capitula **E** habit **F** habitat (black arrow shows a capitulum amongst a tuft of *Festuca chrysophylla*). Pictures by Joel Calvo at the *locus classicus* (between Machuca and El Tatio, San Pedro de Atacama, Antofagasta, Chile).

*Senecio festucoides* grows amongst tufts of *Festuca chrysophylla* Phil. (Poaceae) [= *F. orthophylla* Pilg. according to Ospina et al. (2013)] (Fig. 1F); indeed, the tufts provide support for *S. festucoides* stems, which are not self-supporting. If the new species is not in flower, it is difficult to detect because its leaves are easily confused with those of *Festuca* (Fig. 1C, D). The following species were observed in the same habitat: *Astragalus minimus* Vogel (Leguminosae), *Mulinum crassifolium* Phil. (Apiaceae), *Parastrephia quadrangularis* (Meyen) Cabrera (Compositae), *Pycnophyllum tetrastichum* J.Rémy (Caryophyllaceae), *Senecio scorzonerifolius*, and *Werneria glaberrima* Phil. (Compositae).

#### Phenology.

Collected in flower from January to March.

#### Etymology.

The epithet *festucoides* refers to the conspicuous resemblance of the leaves to those of *Festuca chrysophylla*, amongst which the new species grows.

#### Conservation status.

Thus far, the new species is only known from two locations and has an extent of occurrence of ca. 2000 km^2^. This would fit the category Endangered (EN) according to the B1a criteria (IUCN 2012). However, it is preliminarily assigned to the category Near Threatened (NT) considering that further data on distribution and population dynamics are essential to firmly establish that the species is facing a very high risk of extinction in the wild.

#### Discussion.

**Figure 2.**
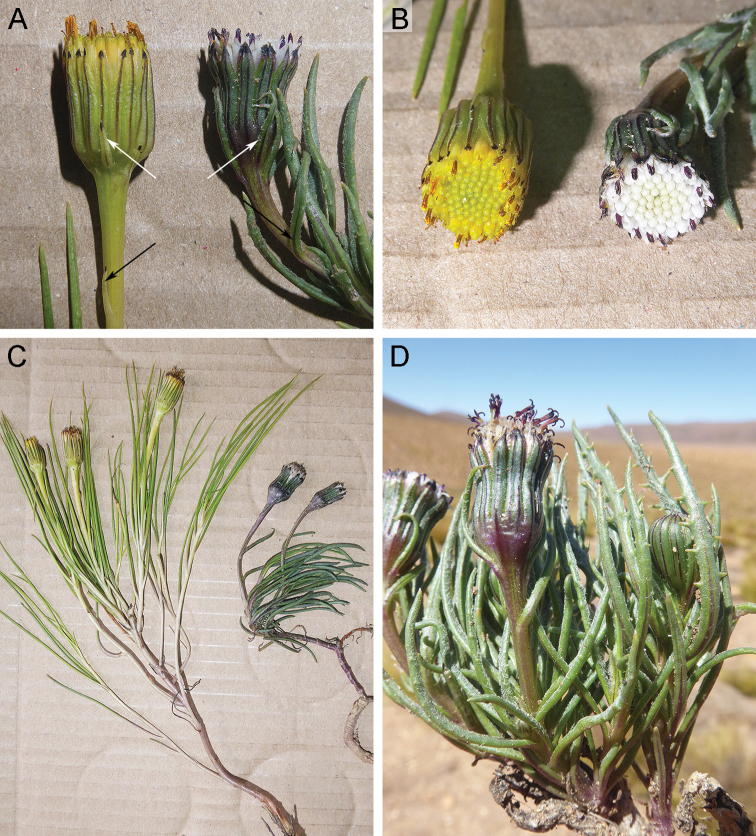
**A–C***Senecio festucoides* (left hand; *Calvo 8120*) and *S. scorzonerifolius* (right hand; *Calvo 8114*) **D***Senecio scorzonerifolius* (*Calvo 8114*) **A** capitula (white arrows show the supplementary bracts; black arrows show the peduncle bracts) **B** florets **C** habit **D** leaves and capitula (notice leaf dimorphism). Pictures by Joel Calvo.

**Figure 3.**
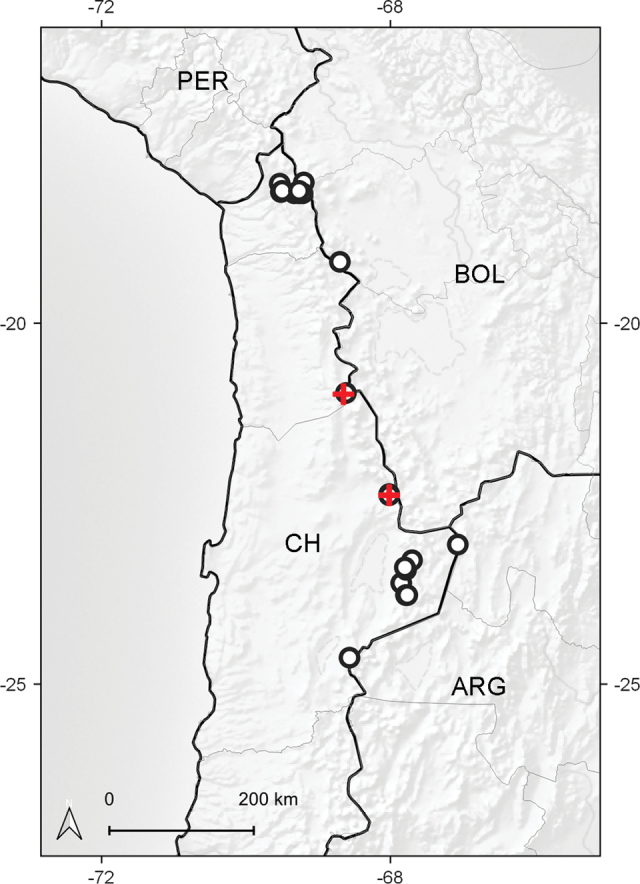
Distribution area of *Senecio festucoides* (red cross) and *S. scorzonerifolius* (open circle) in Chile. Abbreviations: ARG (Argentina), BOL (Bolivia), CH (Chile), and PER (Peru).

*Senecio festucoides* shows morphological affinities with the sympatric species *S. scorzonerifolius* (Fig. 3), which is known from southern Peru, western Bolivia, northwestern Argentina, and northern Chile. Although these species have a similar appearance, they belong to different subgroups within the discoid caespitose Andean *Senecio*, i.e. the new species is a member of the subgroup displaying yellow corollas and yellowish anthers and style branches whereas *S. scorzonerifolius* belongs to the subgroup with white corollas and blackish anthers and style branches (Fig. 2B). They also differ in leaf indumentum (glabrous in *S. festucoides* vs. densely to barely arachnoid, rarely almost glabrous in *S. scorzonerifolius*), peduncle bract type (linear bracts up to 6 mm long in *S. festucoides* vs. leaf-like bracts up to 30 mm long that gradually decrease in size upwards in *S. scorzonerifolius*; see Fig. 2A), involucral bract number and length ((17–)21, 10–11 mm in *S. festucoides* vs. (9)13–15, 8–9 mm in *S. scorzonerifolius*), supplementary bract length (3.9–6 mm, a third as long as the involucral bracts in *S. festucoides* vs. 6–8 mm, a half to two thirds as long as the involucral bracts in *S. scorzonerifolius*; see Fig. 2A), and achene indumentum (minutely papillose in *S. festucoides* vs. papillose in *S. festucoides*). Moreover, *S. festucoides* has longer stems and does not develop crowded tufts as *S. scorzonerifolius* usually does (Fig. 2C, D). In living plants, their leaf color is also different (yellowish green in *S. festucoides* vs. dark green in *S. scorzonerifolius*; see Fig. 2C).

The leaf shape of the new species might lead any botanist to confuse it with *S. bolivarianus* Cuatrec., a species endemic to Peru known from Ancash to Moquegua (Beltrán and Roque 2015). They can be readily differentiated by the abaxial leaf surface (densely silky-villous except for the midrib in *S. bolivarianus* vs. glabrous in *S. festucoides*), leaf margin (revolute in *S. bolivarianus* vs. flat in *S. festucoides*), leaf base (broadened into a sheath-like base that bears long silky trichomes in *S. bolivarianus* vs. uniform in width and glabrous in *S. festucoides*), and number of supplementary bracts (12–16 in *S. bolivarianus* vs. (3–)5–8 in *S. festucoides*). Moreover, *S. bolivarianus* has linear to narrowly lanceolate leaves rather than narrowly linear as in *S. festucoides*.

It should be noted that *S. festucoides* is characterized by its narrowly linear, entire leaves but we also observed a few specimens displaying both entire and distantly dentate leaves. Such leaf dimorphism is usual in other related species such as *S. digitatus* Phil. (Calvo et al. 2019) and *S. scorzonerifolius* (Cabrera 1985; Fig. 2D).

#### Additional specimens examined.

*Senecio festucoides* (paratypes). **Chile. Antofagasta**: Loa, San Pedro de Atacama, Machuca-El Tatio, ca. 6.2 km al S de El Tatio, 22°23'S, 68°1'W, 5 Mar 2019, *J. Calvo 7925* (SGO); **Tarapacá**: Collahuasi, quebrada San Nicolás, 20°59'S, 68°39'W, 22 Jan 1994, *S. Teillier 3296* (SGO).

*Senecio scorzonerifolius*. **Bolivia. Oruro**: Sabaya, parte alta del Pumire, 19°0'S, 68°25'W, 7 Feb 2019, *J. Calvo 7838* (LPB); cerro Cabaray [Carabaya], faldeos, 19°9'S, 68°42'W, 23 Mar 1982, *C. Villagrán & M.T.K. Arroyo 4209* (CONC); **Potosí**: cordillera Kari Kari, aprox. 3.3 km arriba de la laguna San Sebastián, 19°36'S, 65°41'W, 13 Feb 2019, *J. Calvo & M. Zárate 7868* (BOLV); Frías, between laguna Lobato and laguna Chalvira, cordillera Kari Kari, 19°38'S, 65°42'W, 6 Mar 1999, *J.R.I. Wood 14596* (K). **Chile. Antofagasta**: Loa, San Pedro de Atacama, Toconao, quebrada Aguas Blancas, 23°17'S, 67°42'W, 22 Feb 2001, *M. Ackermann 93* (SGO); cerro Nevados de Poquis, ladera SO, 23°4'S, 67°4'W, 9 Apr 1997, *M.T.K. Arroyo, L. Cavieres & A. Humaña 97367* (CONC); cerro Miñiques, 23°46'S, 67°46'W, 9 Mar 1993, *G. Baumann 192* (CONC); quebrada al lado sur del volcán Lascar, 23°23'S, 67°48'W, 14 Feb 1994, *G. Baumann 351* (CONC); Machuca-El Tatio, ca. 4.2 km al S de El Tatio, 22°22'S, 68°1'W, 5 Mar 2019, *J. Calvo 7923* (CONC, SGO); Machuca, 4.1 km antes de la entrada a los géisers del Tatio, 22°23'S, 68°1'W, 27 Feb 2020, *J. Calvo 8114* (SGO); Toconao, 57 km pass, 23°36'S, 67°51'W, 27 Jan 1971, *H. Ellenberg 4230* (US); inter Aguas Calientes et Socaire, 23°46'S, 67°47'W, 1 Feb 1885, *F. Philippi s.n.* (LP, SGO; type material of the later heterotypic synonym *Senecio armeriifolius* Phil.); quebrada Tatio, 15 Feb 1943, *E. Pisano & J. Venturelli 1879* (CONC, SGO); laguna de Miñique, entre los cerros Miñique y Miscanti, 23°46'S, 67°47'W, 24 Feb 1943, *E. Pisano & J. Venturelli 1968* (SGO); trayecto entre Talabre y laguna Lejía, 23°25'S, 67°47'W, 2 Apr 1998, *C. Villagrán, F. Hinojosa & C. Latorre 9315* (CONC); Taltal, cord. volcán Llullaillaco, 24°38'S, 68°34'W, Feb 1926, *E. Werdermann 1029* (CONC); **Arica-Parinacota**: camino entre Putre y Pacollo, 18°11'S, 69°31'W, 17 Apr 1984, *M.T.K. Arroyo 84-887* (CONC); rt. A23 from rt. 11 NW to Tacora, near intersection of rt. A23 & A125, slopes of Co. de Tarapacá, 17.6 km from rt. 11, 18°4'S, 69°32'W, 7 Mar 2014, *V.A. Funk, M. Diazgranados & J.M. Bonifacino 13111* (US); camino de Putre a Chucuyo, km 8, 18°10'S, 69°30'W, 12 Feb 1964, *C. Marticorena, O. Matthei & M. Quezada 179* (CONC); lagunas de Cotacotani, 18°12'S, 69°13'W, 13 Feb 1964, *C. Marticorena, O. Matthei & M. Quezada 236* (CONC); cerro Guane-Guane, ladera W, 18°10'S, 69°16'W, 18 Mar 2015, *A. Moreira-Muñoz & F. Luebert 2423* (SGO); portezuelo de Putre, laderas de los cerros, 18°12'S, 69°20'W, 6 May 1972, *M. Ricardi, E. Weldt & M. Quezada 237* (CONC); Parinacota, 18°12'S, 69°16'W, 29 Mar 1961, *M. Ricardi, C. Marticorena & O. Matthei 291* (CONC); cerros de Parinacota, 28 Feb 1948, *F. Sudzuki 486* (SGO); Caquena, 18°3'S, 69°12'W, 1 Feb 1970, *O. Zöllner 5321* (CONC); **Tarapacá**: Iquique, Collahuasi (Ujina), 20°58'S, 68°37'W, 24 Mar 1992, *B.J. Ruthsatz 8429* (CONC). **Peru. Moquegua**: minera Quellaveco, 17°6'S, 70°36'W, 27 Jan 1971, *ESCO 7240* (US).

### Key to the discoid caespitose *Senecio* species from northern Chile

The dwarf shrubs developing erect stems are excluded (e.g. *Senecio puchei* Phil., *S. socompae* Cabrera, *S. trifurcifolius* Hieron.). *Senecio festucoides* is not a strictly caespitose species but it is included in this informal group on the basis of the taxonomic placement of its morphologically similar species. As pointed out in Calvo et al. (2019), the color of the corollas, anthers, and style branches has taxonomic value within this group and is readily noticeable in living plants. However, a careful study of these characters on dried specimens is required to avoid misidentifications.

**Table:** 

1	Leaves sparsely tomentose to lanate	**2**
–	Leaves glabrous	**6**
2	Leaves 4−8 cm long, narrowly linear (leaf width/length ratio 0.01−0.05); achenes papillose	***S. scorzonerifolius***
–	Leaves 1−3 cm long, widely linear (leaf width/length ratio 0.08−0.13), elliptic, oblanceolate, or spatulate; achenes glabrous or silky-pubescent	**3**
3	Achenes glabrous; corollas yellow; anthers and style branches yellowish	**4**
–	Achenes silky-pubescent; corollas white; anthers and style branches blackish	**5**
4	Leaves entire, indumentum densely lanate	***S. evacoides***
–	Leaves pinnatifid to pinnatipartite, indumentum laxly lanate	***S. helgae***
5	Leaf laminas widely linear to slightly spatulate, base progressively narrowed, apex rather acute and usually with a callus-like tip, indumentum arachnoid	***S. digitatus***
–	Leaf laminas ovate to suborbicular, base petioliform, apex obtuse and unadorned, indumentum pilose	***S. pygmophyllus***
6	Leaves 1–2-pinnatisect	***S. jarae***
–	Leaves entire or distantly dentate	**7**
7	Leaves 4–8 cm long, narrowly linear	**8**
–	Leaves 0.5–3.5(–5) cm long, linear-oblong, oblanceolate, or spatulate	**9**
8	Involucral bracts (17–)21; corollas yellow; anthers and style branches yellowish	***S. festucoides***
–	Involucral bracts (9)13–15; corollas white; anthers and style branches blackish	***S. scorzonerifolius***
9	Stems hypogeous; leaf laminas spatulate, 3.6–7 mm wide; achenes long-pilose (trichomes 0.3–0.35 mm long)	***S. toconaoensis***
–	Stems epigeous; leaf laminas linear-oblong to oblanceolate, 1–5 mm wide; achenes glabrous or with scattered short trichomes (0.1–0.2 mm long)	**10**
10	Involucre 7.5–10 mm long; involucral bracts 12–15	***S. algens***
–	Involucre 4–5 mm long; involucral bracts 8(–9)	***S. humillimus***

## Supplementary Material

XML Treatment for Senecio festucoides
